# Porous TiO_2_ Assembled from Monodispersed Nanoparticles

**DOI:** 10.1186/s11671-016-1372-2

**Published:** 2016-03-22

**Authors:** Xu Liu, Weijie Duan, Yan Chen, Shihui Jiao, Yue Zhao, Yutang Kang, Lu Li, Zhenxing Fang, Wei Xu, Guangsheng Pang

**Affiliations:** State Key Laboratory of Inorganic Synthesis and Preparative Chemistry, College of Chemistry, Jilin University, Changchun, Jilin 130012 People’s Republic of China

**Keywords:** TiO_2_ nanoparticles, Porous TiO_2_, Assemblies, Photodegradation

## Abstract

Porous TiO_2_ were assembled by evaporating or refluxing TiO_2_ colloid, which was obtained by dispersing the TiO_2_ nanoparticles with a crystallite size (*d*_XRD_) of 3.2 nm into water or ethanol without any additives. Porous transparent bulk TiO_2_ was obtained by evaporating the TiO_2_-C_2_H_5_OH colloid at room temperature for 2 weeks, while porous TiO_2_ nanospheres were assembled by refluxing the TiO_2_-H_2_O colloid at 80 °C for 36 h. Both of the porous TiO_2_ architectures were pore-size-adjustable depending on the further treating temperature. Porous TiO_2_ nanospheres exhibited enhanced photocatalysis activity compared to the nanoparticles.

**Electronic supplementary material:**

The online version of this article (doi:10.1186/s11671-016-1372-2) contains supplementary material, which is available to authorized users.

## Background

Size plays an outstanding role in determining the properties of materials. After several decades of intense research on size effects of single nanoparticle or small nanoparticle clusters, the trend goes in the direction of meso- and macroscale structures consisting of nanoscale building blocks [1]. Nanoparticles, which possess the characters of small size and large surface area, have received widely attention due to their unique properties of optics, magnetics, electrics and catalysis [2–5]. To expand the research on nanoparticles, there has been growing interest in constructing hierarchical structures consisting of aggregated nanoparticles [6–8]. The combination of size effects and collective properties gives rise to fascinating opportunities for the creation of unprecedented materials. It is important to control the organization and dispersion states to assemble nanomaterials [9–11]. For example, the homogeneous and disordered assembly of densely packed nanoparticles can result in bulk transparent film-like objects through gradually evaporating the high-quality homogeneous nanoparticle colloid [12, 13]. The high transmittance benefits from the homogeneous particles, which are quite small and do not scatter visible light. The homogeneous assembly of densely packed nanoparticles results in a narrow range of pore sizes that are highly dependent on the size and uniformity of the nanoparticles [14, 15].

As a most promising photocatalyst, anatase TiO_2_ has been widely investigated to improve its photocatalytic performance. Porous TiO_2_ has performed much higher photocatalytic activity than solids due to their stronger adsorption to substrates and/or higher light-harvesting ability [16–19]. The void space can modulate the refractive index, lower the density, increase the active area for catalysis and improve the catalysts’ ability to withstand cyclic changes in volume. Furthermore, void space in hollow structures can be used to encapsulate and control release of sensitive materials such as drugs and cosmetics. TiO_2_ nanomaterials with different morphologies have been reported, and it is still a challenge to assemble TiO_2_ nanoparticles (TiO_2_-NPs) to form a porous structure [20–22].

In our study, monodispersed anatase TiO_2_-NPs were prepared by the hydrolysis of tetrabutyltitanate (TBT) in a cyclohexane/ethanol medium. The assembly of TiO_2_-NPs was investigated under different conditions and resulted in transparent bulk TiO_2_ and porous TiO_2_ nanospheres (TiO_2_-NSs).

## Methods

### Preparation of TiO_2_-NPs

All of the chemicals were of analytic grade and used without further purification. We prepared TiO_2_ nanoparticles by the hydrolysis of tetrabutyltitanate (TBT) in a cyclohexane (C_6_H_12_)/ethanol (C_2_H_5_OH) medium under refluxing conditions as reported [23]. Briefly, 36 mL of TBT was dissolved in the mixed solvents (90 mL of ethanol and 90 mL of cyclohexane), and 9 mL of HCl (TBT/HCl (*v*/*v*) = 1:0.25) was added to it. The final homogeneous solution was refluxed at 67 °C for 10 h and then cooled to room temperature. To precipitate the TiO_2_-NPs, 600 mL of ethanol was added dropwise to the solution under stirring. After standing overnight, a white precipitate was harvested by centrifugation. The powder was obtained by washing with ethanol three times and dried in air at room temperature. (Additional file 1 shows the X-ray diffraction (XRD) pattern, transmission electron microscopy (TEM) image and the size distribution histogram of TiO_2_-NPs).

### Preparation of the Porous Transparent Bulk TiO_2_

Ten milligrams of freshly prepared TiO_2_-NPs was added to 10 mL of ethanol in a 1000-mL beaker covered with plastic wrap. After standing for 2 days, the turbid suspension gradually changed to a transparent TiO_2_-C_2_H_5_OH colloid. Porous transparent bulk TiO_2_ was obtained by evaporating the TiO_2_-C_2_H_5_OH colloid at room temperature for 2 weeks. To investigate the thermal stability, porous transparent bulk TiO_2_ was heated at different temperatures for 2 h with a heating rate of 5 °C/min in air.

### Preparation of TiO_2_-NSs

The freshly prepared TiO_2_-NPs were dispersed into deionized water to form a TiO_2_-H_2_O colloid. Typically, 100 mL of a 10-g/L TiO_2_-H_2_O colloid was heated at 80 °C for 36 h. Then, the precipitate was collected by centrifugation, washed with deionized water several times, and dried in air at room temperature. The thermal stability of the TiO_2_-NSs was studied by heating at different temperatures for 2 h with a rate of 5 °C/min in air.

### Characterization

Powder XRD patterns were recorded on a PANalytical B.V. Empyrean X-ray diffractometer with graphite-filtered Cu *Kα* radiation at 40 kV and 40 mA. A SU8020 electron microscope was applied to get scanning electron microscopy (SEM) images. High-resolution transmission electron microscopy (HRTEM) was performed on a Tecnai G2 S-TWIN F20 at an accelerating voltage of 200 kV. Before being studied by HRTEM, porous transparent bulk TiO_2_ specimens were fabricated on an FEI Helios NanoLab 600i FIB/SEM dual-beam system. Infrared (IR) spectra of the samples were recorded on a Bruker IFS-66V/S FT-IR spectrometer with a resolution of 2 cm^−1^. Thermogravimetric analysis (TG) was carried out on a NETZSCH STA 449 F3 with a heating rate of 20 K/min from room temperature to 800 °C. Brunauer-Emmett-Teller (BET) measurements were performed via the nitrogen adsorption method on a Micromeritics ASAP 2420, and UV-Vis absorption was measured on a UV-2450 spectrophotometer.

### Photodegradation

The photocatalytic activity of TiO_2_-NPs and TiO_2_-NSs was tested by measuring the degradation rate of methylene blue (MB) under UV light irradiation. Typically, 50 mg of the as-prepared TiO_2_ catalyst was added to 50 mL of an aqueous solution containing 10 mg/L MB. The mixture was magnetically stirred in the dark at ambient temperature for 1 h to achieve adsorption-desorption equilibrium of TiO_2_ with MB followed by exposure to UV light from a 125-W high-pressure mercury vapour lamp. Five milliliters of the suspension was extracted every 10 min, and the suspended solid was immediately separated by centrifugation. UV-Vis absorption spectra were measured to monitor the concentration of MB remaining in the aqueous system. For comparison, Degussa P25 was adopted to investigate the photocatalytic activity under the same condition.

## Results and Discussion

### The Assembly of Porous Transparent Bulk TiO_2_

**Fig. 1:**
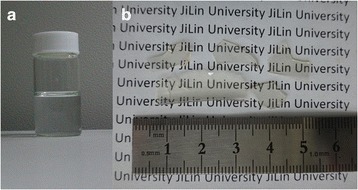
Digital photographic images of (**a**) TiO_2_-C_2_H_5_OH colloid; (**b**) porous transparent bulk TiO_2_

**Fig. 2:**
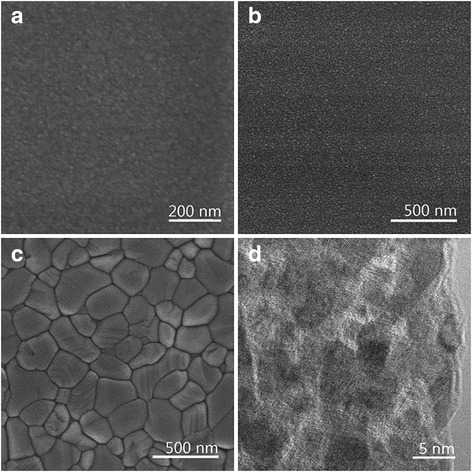
SEM and TEM images of porous transparent bulk TiO_2_. **a** SEM image of the untreated porous transparent bulk TiO_2_. **b** SEM image of porous transparent bulk TiO_2_ heating at 500 °C. **c** SEM image of porous transparent bulk TiO_2_ heating at 800 °C. **d** TEM image of porous transparent bulk TiO_2_

When freshly prepared TiO_2_-NPs with a crystallite size (*d*_XRD_) of 3.2 nm (see Additional file 1) were dispersed in ethanol, the turbid suspension gradually became a transparent TiO_2_-C_2_H_5_OH colloid after standing for 2 days. The digital photographic image is shown in Fig. 1a. After evaporating at room temperature, the colloid slowly condensed and formed porous transparent objects with size of 1–3 cm as shown in Fig. 1b. The IR spectrum and TG analysis of the porous transparent bulk TiO_2_ are present in Additional file 1: Figure S4 and Figure S5. IR spectrum (Additional file 1: Figure S4) shows the absorption peaks corresponding to the surface hydroxy group (Ti-O-H) and/or water molecules (O-H stretching mode at ~3200 cm^−1^ and O-H bending mode at ~1620 cm^−1^). TG analysis (Additional file 1: Figure S5) shows the weight losses from room temperature to 200 °C or higher, which indicates the loss of physically adsorbed water and the surface hydroxy groups. The hydrated layer formed around the nanoparticles and their hydrophilic nature leads to the formation of homogeneous and closely packed nanocrystals [12]. The nanoparticles dispersed in ethanol were homogeneously assembled into bulk materials after the evaporation of ethanol without the formation of inhomogeneous aggregates. During evaporation, TiO_2_-NPs in the colloid were drawn to the meniscus by convective transport; capillary forces pushed the organization of close-packed structures [24]. Figure 2a shows the SEM image of the smooth surface; no grain boundaries or cracks were observed on micrometre scale of the bulk objects. The micropores in porous transparent bulk TiO_2_ are confirmed by TEM (Fig. 2d) and N_2_ physisorption measurements and are formed in the interspaces of the densely packed TiO_2_-NPs.

**Fig. 3:**
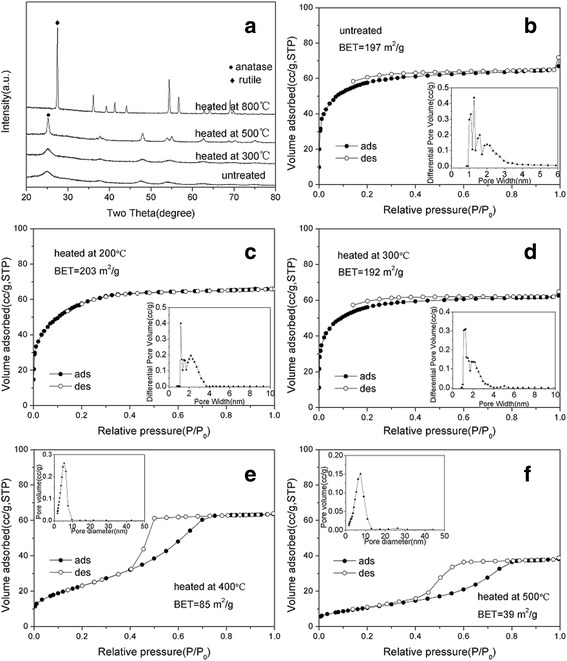
**a** XRD patterns of porous transparent bulk TiO_2_. **b**–**f** N_2_ adsorption-desorption isotherms and pore size distribution curves of porous transparent bulk TiO_2_ heated at various temperatures

Figure 3b shows the N_2_ adsorption-desorption isotherms and the corresponding pore size distribution curves of porous transparent bulk TiO_2_. The curve indicates a sharp uptake at low relative pressures and gradually increasing uptake at higher relative pressures. The adsorption isotherm is of type I according to the IUPAC classification, which indicates the presence of micropores [25]. The specific surface area of porous transparent bulk TiO_2_ was 197 m^2^/g, and the main pore size was located around 1.3 nm. This result agrees well with the homogenous and disordered assembly of nanoparticles in the porous transparent bulk TiO_2_.

The as-prepared porous transparent bulk TiO_2_ was thermally stable up to 500 °C. No grain boundaries or cracks on micrometre scale were observed (Fig. 2b). After heating at 800 °C for 2 h, boundaries were observed on the surface of the objects using SEM, as shown in Fig. 2c, and the phase was transformed from anatase to rutile during the thermal treatment (Fig. 3a). The BET specific surface decreased, and the pore size gradually increased with heating temperature. The shape of the isotherm of the sample heated above 400 °C turns to type IV with type H2 hysteresis as shown in Fig. 3e, f. This result is consistent with the transformation of pore size from microporosity to mesoporosity as given in Table 1. After heating at 500 °C, the BET specific surface area decreased to 39 m^2^/g, and the pore size was 6.9 nm, which indicates that the pore size of porous transparent bulk TiO_2_ was adjustable from microporous to mesoporous via thermal treatment.

**Table 1:** Specific surface area and pore size of porous transparent bulk TiO_2_ heated at various temperatures

Sample	Specific surface area (m^2^/g)	Average pore size (nm)
Untreated	197	1.3
Heated at 200 °C	203	1.6
Heated at 300 °C	192	1.8
Heated at 400 °C	85	5.4
Heated at 500 °C	39	6.9

### The Assembly of TiO_2_-NSs

**Fig. 4:**
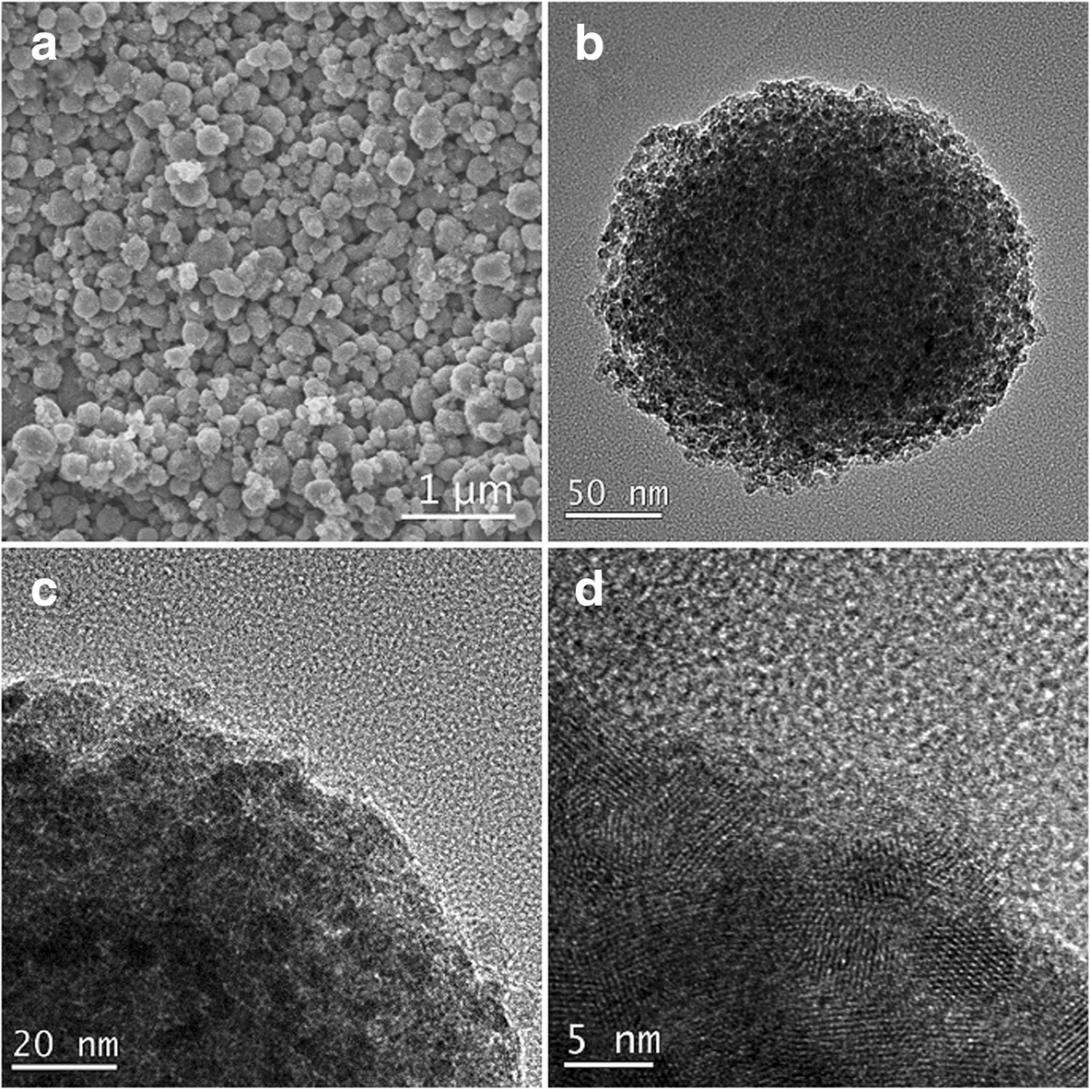
**a** SEM image. **b**, **c** TEM images. **d** HRTEM image of assembled TiO_2_-NSs

Freshly prepared TiO_2_-NPs can be dispersed into deionized water to form a colloid without any additives [23], and porous TiO_2_-NSs can be prepared by heating 10 g/L TiO_2_-H_2_O colloid at 80 °C for 36 h. The SEM image of the TiO_2_-NSs is shown in Fig. 4a, and the results indicate that the nanoparticles assembled into spheres due to the heat treatment. The assembled spheres had a spherical diameter of 100–270 nm. Detailed information of a single TiO_2_-NS was provided by TEM (Fig. 4b). The nanospheres had a rough surface and were assembled by nanoparticles (Fig. 4c). In addition, the porous interior is shown in Fig. 4b, c. As revealed in the TEM images, the spheres were composed of TiO_2_-NPs.

**Fig. 5:**
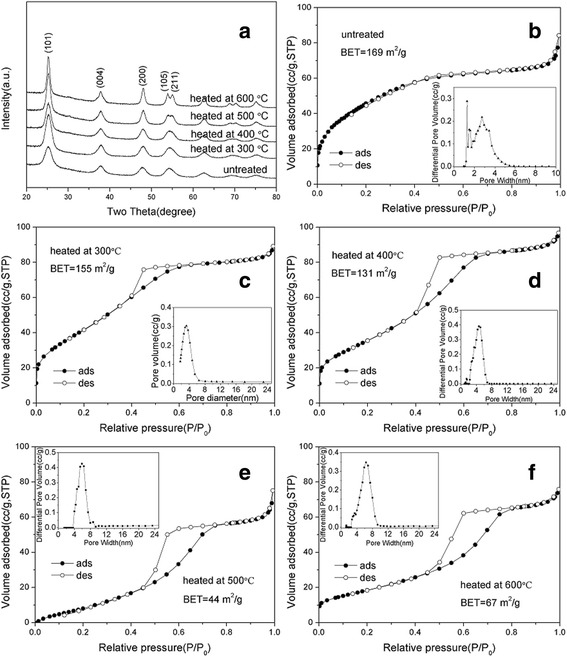
**a** XRD patterns of TiO_2_-NSs. **b**–**f** N_2_ adsorption-desorption isotherms and the pore size distribution curves of TiO_2_-NSs heated at various temperatures

The HRTEM micrograph shown in Fig. 4d indicates that each nanoparticle was fully crystallized with a grain size (*d*_TEM_) of *ca.* 5 nm. The result is consistent with the analysis of XRD data of untreated TiO_2_-NSs shown in Fig. 5a, which is assigned to anatase TiO_2_ (JCPDS card no. 21-1276) with crystallite size (*d*_XRD_) of 4.6 nm. Heating treatment in the water encouraged the grain growth due to the Ostwald ripening mechanism and resulted in a larger crystallite size of 4.6 nm compared to the original size of 3.2 nm.

The pore size of the TiO_2_-NSs was confirmed by N_2_ physisorption measurements. Figure 5b–f shows the N_2_ adsorption-desorption isotherms (type IV) [25] and the corresponding pore size distribution curves of the samples heated under different temperatures. The BET specific surface area of the untreated sample was 169 m^2^/g, and the average pore size was 1.9 nm (Fig. 5b), which was larger than the transparent bulk TiO_2_ due to the bigger crystallite size of the units. After heating at different temperatures, the BET specific surface area and pore size of the assembled nanospheres changed gradually, which can also be ascribed to the increase of the crystallite size, as shown in Table 2. The TiO_2_-NSs heated at 600 °C exhibited the largest pore size (6.4 nm), and the untreated TiO_2_-NSs exhibited the largest BET specific surface area (169 m^2^/g).

**Table 2:** Specific surface area, pore size and crystallite size of TiO_2_-NSs heated at various temperatures

Sample	Specific surface area (m^2^/g)	Average pore size (nm)	Crystallite size (*d*_XRD_) (nm)
Untreated	169	1.9	4.6
Heated at 300 °C	155	3.4	5.1
Heated at 400 °C	131	4.7	5.8
Heated at 500 °C	44	5.9	6.9
Heated at 600 °C	67	6.4	9.6

### Photocatalytic Activities of TiO_2_-NPs and TiO_2_-NSs

**Fig. 6:**
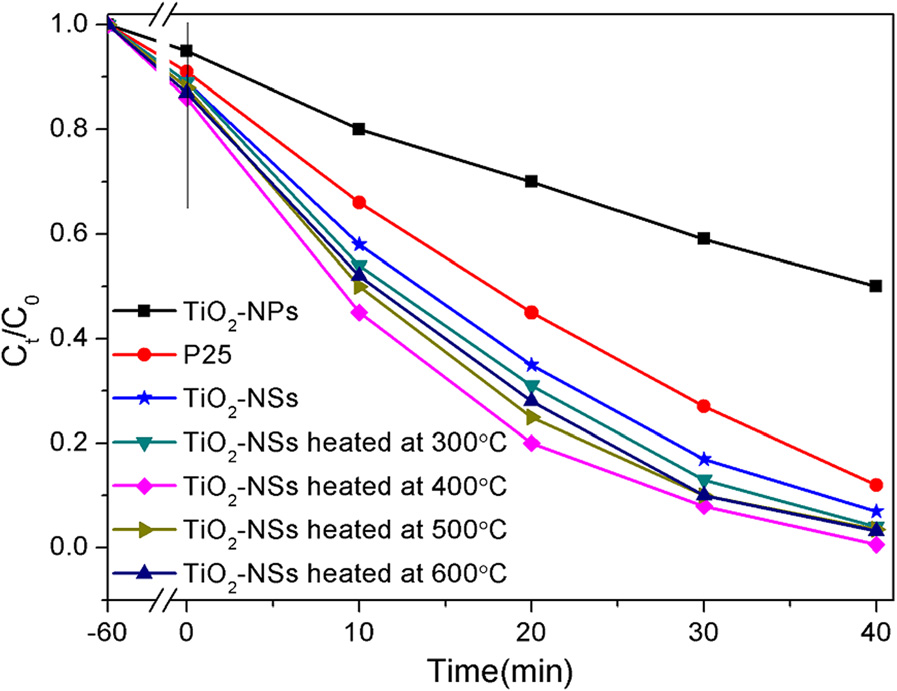
Photodegradation curves. Photodegradation of MB solutions using TiO_2_-NPs (untreated), commercial P25 and TiO_2_-NSs heated at various temperatures as photocatalysts under UV irradiation

The photocatalytic activities of TiO_2_-NPs and TiO_2_-NSs were evaluated by the degradation of MB under UV light irradiation. As shown in Fig. 6, the photocatalytic result indicates that the photocatalytic performance was substantially improved after the assembly of TiO_2_-NPs into TiO_2_-NSs. MB was almost completely removed in 40 min by the TiO_2_-NSs heated at 400 °C, which exhibit superior performance among the photocatalysts we studied, including P25.

The number of effective active sites on the surface is an important character of the activity of a photocatalyst, which would be affected by many factors. Photocatalysts with smaller particle size usually exhibit higher activity. The reason may be that when the particle size is smaller, the specific surface area is larger, and more photocatalysis active sites are available. Whereas, when the particle size is too small, most of the electrons and holes are generated close to the surface and surface recombination is faster than interfacial charge carrier transfer processes. It is reported that when the particle size of TiO_2_ is smaller than 10 nm, the photocatalytic activity will decrease [26, 27]. But the crystallite size (*d*_XRD_) of as-prepared TiO_2_-NSs in this work was 4.6 nm which is far less than 10 nm. In addition, the spheres were assembled from nanoparticles by the heat treatment. Surface states play an important role in the recombination process of electron and hole [28]. We believe that the effective contact among nanoparticles affected the surface states which could reduce the recombination chance of electron and hole. Therefore, the photocatalytic activity of TiO_2_-NSs improved compared to TiO_2_-NPs.

Moreover, the defect in nano semiconductor photocatalysts plays an important role in photocatalysis [29–31]. The photocatalytic enhancement upon the calcination is attributed to the fact that increasing calcination temperature leads to a decrease in the concentration of bulk defects in TiO_2_ due to the increased crystallinity evidenced by XRD (Fig. 5a), thus reducing the recombination of photogenerated electrons and holes. As can be seen from Fig. 6 and Table 2, the specific surface area of the TiO_2_-NSs samples decreases with increasing calcination temperature, but actually it should be understood as the increase of the specific photocatalytic activity (per unit surface area), that leads the photocatalytic activity of TiO_2_-NSs heated at 400 °C higher than that of uncalcined TiO_2_-NSs and other calcinated TiO_2_-NSs.

## Conclusions

Porous transparent bulk TiO_2_ was formed upon slow evaporation of TiO_2_-C_2_H_5_OH colloid. The crystalline building blocks are clearly distinguishable in the free-standing macroscopic bodies. Porous TiO_2_-NSs were assembled by refluxing the TiO_2_-H_2_O colloid and exhibited enhanced photocatalysis activity compared to the nanoparticles. Both of the porous TiO_2_ architectures were pore-size-adjustable depending on the further treating temperature. The current strategy and resulting materials have potentials of designing a variety of bulk objects. The controlled assembly of nanoparticles might open up the pathway to a variety of macroscopic materials with hierarchical architectures and complex morphologies.

## Additional File

Additional file 1: Supporting information. **Figure S1.** XRD patterns of TiO_2_-NPs. **Figure S2.** TEM image of TiO_2_-NPs. **Figure S3.** Size distribution histogram of TiO_2_-NPs. **Figure S4.** IR spectrum of the porous transparent bulk TiO_2_. **Figure S5.** TG analysis of the porous transparent bulk TiO_2_.
