# Supplementary material for: Porous TiO2 Assembled from Monodispersed Nanoparticles
Source: Nanoscale Res Lett. 2016 Mar 22;11:159. doi: 10.1186/s11671-016-1372-2 (PMC4801840; doi:10.1186/s11671-016-1372-2)
Supplement: Additional file 1: — Supporting information. Figure S1. XRD patterns of TiO2-NPs. Figure S2. TEM image of TiO2-NPs. Figure S3. Size distribution histogram of TiO2-NPs. Figure S4. IR spectrum of the porous transparent bulk TiO2. Figure S5. TG analysis of the porous transparent bulk TiO2. [file 11671_2016_1372_MOESM1_ESM.docx]

**Suporting Information**

**Porous TiO_2_ assembled from monodispersed nanoparticles**

Xu Liu, Weijie Duan, Yan Chen, Shihui Jiao, Yue Zhao, Yutang Kang, Lu Li, Zhenxing Fang, Wei Xu, and Guangsheng Pang*

State Key Laboratory of Inorganic Synthesis and Preparative Chemistry, College of Chemistry, Jilin University, Changchun, Jilin 130012, P. R. of China

E-mail: [panggs@jlu.edu.cn](mailto:panggs@jlu.edu.cn)


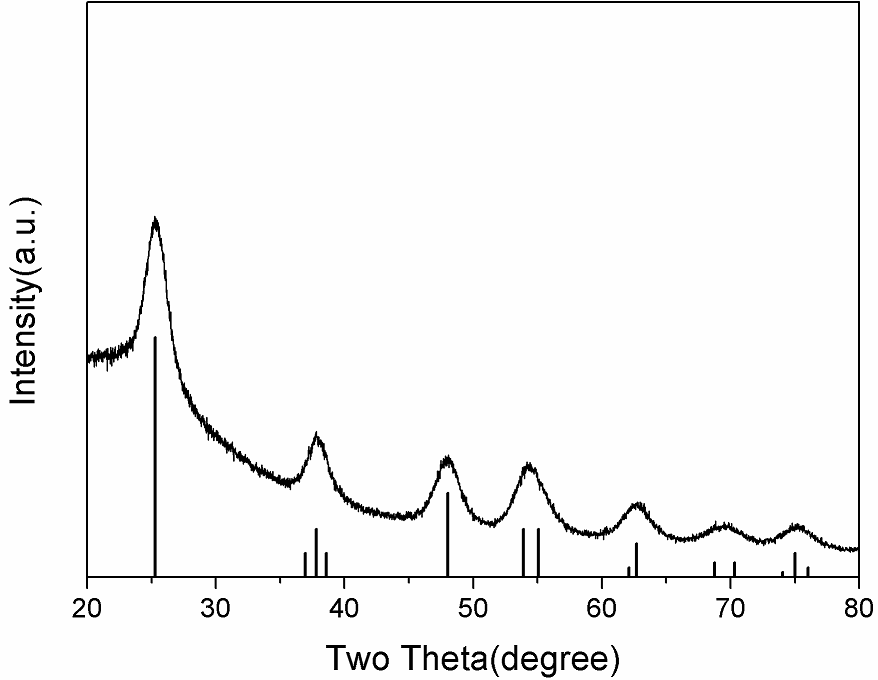


**Figure S1.** XRD patterns of TiO_2_-NPs

Figure S1 shows the XRD patterns of the as-prepared TiO_2_ nanoparticles (TiO_2_-NPs) products synthesized under refluxing conditions. All the diffraction peaks were well indexed to the pure anatase phase of TiO_2_ (JCPDS No. 21-1272). The crystallite size (*d*_XRD_) was 3.2 nm, which was determined from the diffraction peak broadening via the Scherrer equation.


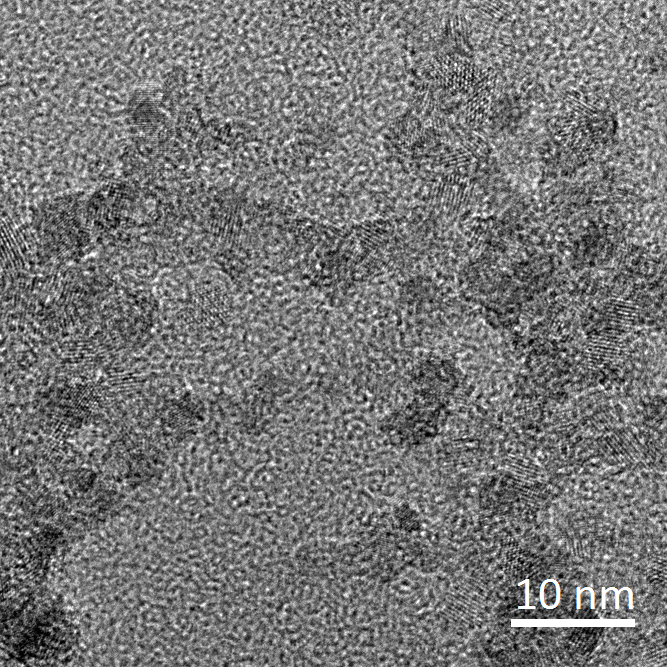


**Figure S2.** TEM image of TiO_2_-NPs

As shown in figure S2, the as-prepared anatase TiO_2_ product is well-dispersed nanoparticles. The grain size (*d*_TEM_) determined by TEM was 3.0 nm based on ca. 100 particles in the sample (the size distribution histogram is shown in figure S3), which is in good agreement with the XRD result.


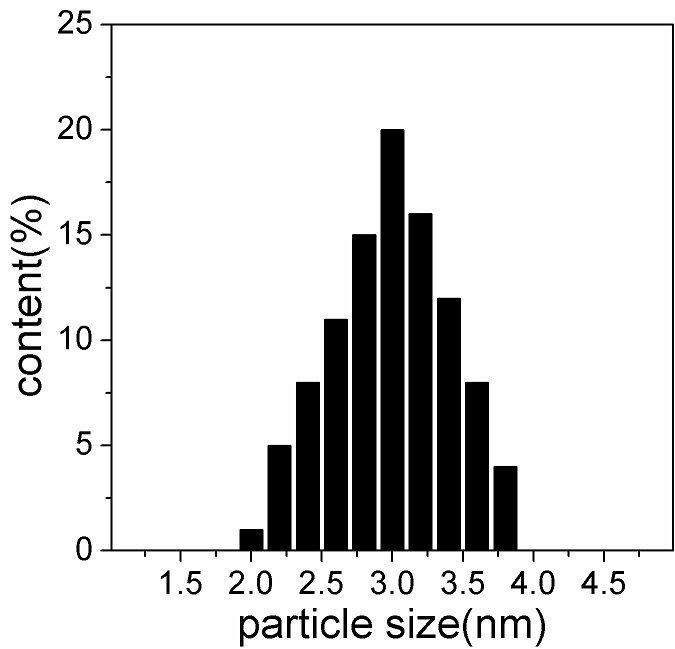


**Figure S3.** Size distribution histogram of TiO_2_-NPs


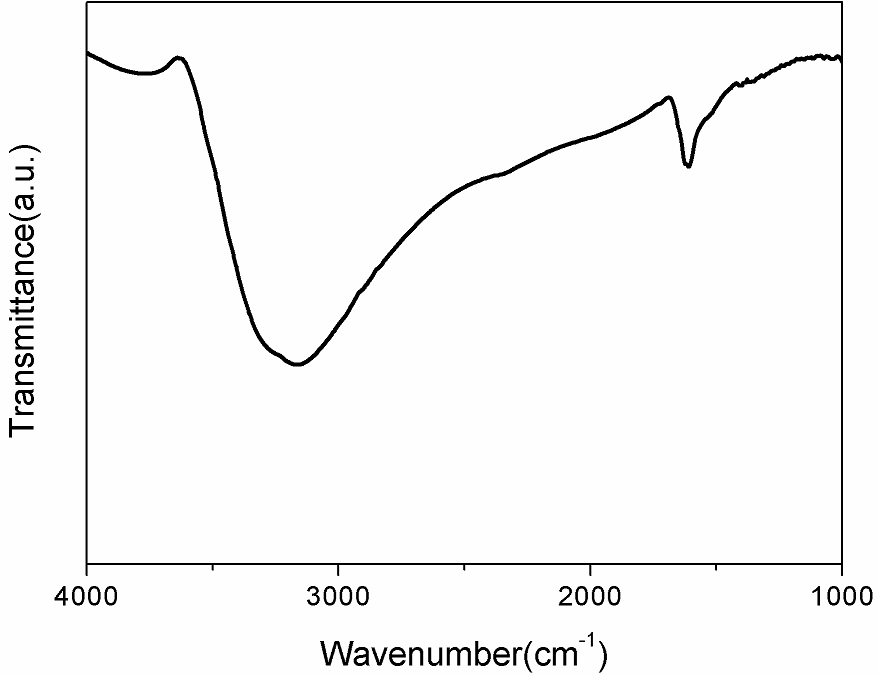


**Figure S4.** IR spectrum of the porous transparent bulk TiO_2_


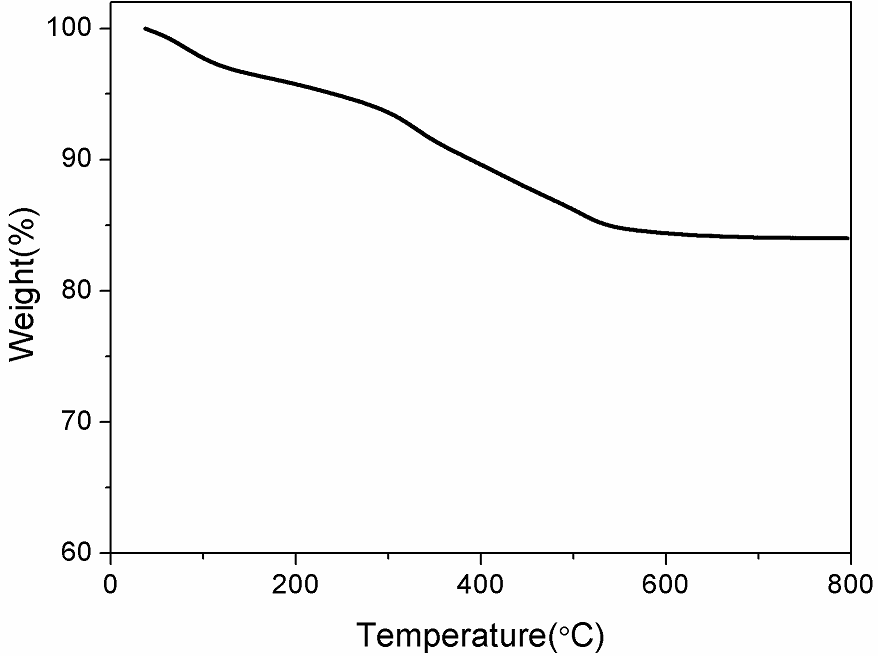


**Figure S5.** TG analysis of the porous transparent bulk TiO_2_
